# Emerging Roles of Primary Cilia in Glioma

**DOI:** 10.3389/fncel.2019.00055

**Published:** 2019-02-20

**Authors:** Matthew R. Sarkisian, Susan L. Semple-Rowland

**Affiliations:** ^1^Department of Neuroscience, McKnight Brain Institute, University of Florida College of Medicine, Gainesville, FL, United States; ^2^Preston A. Wells, Jr. Center for Brain Tumor Therapy, McKnight Brain Institute, University of Florida College of Medicine, Gainesville, FL, United States

**Keywords:** glioblastoma, cilium, brain cancer, temozolomide, ciliary signaling

## Abstract

Primary cilia are microtubule-based organelles that are typically present on cells during the G0 or G1-S/G2 phases of the cell cycle. Recent studies of glioblastoma (GBM) biopsies, a brain tumor that is notorious for its aggressive growth and resistance to treatment, show that many cells in the tumor lack cilia. At this point, it remains unclear whether primary cilia promote or suppress glioma tumorigenesis. In this review, we will discuss the different roles that have been proposed for primary cilia in glioma and how cilia may contribute to the resistance of these tumors to current therapies.

## Introduction

Primary cilia are microtubule-based organelles that relay signals to the cell on which they reside and release signals into the cellular microenvironment (Wood and Rosenbaum, 2015; Garcia et al., 2018), but their functions in glioma remain unclear. To our knowledge Schuster (1964) were the first to report the presence of ciliated fibroblasts in a human brain tumor. Despite detecting cilia bearing long axonemes, these investigators concluded that “it is difficult to imagine these cilia fulfilling any useful function, confined and atypical as they are.” A few years later, Tani and Ametani (1970) described ciliated cells in human gliomas and concluded that the cilia “…might be called vestigial; merely formed because of an inherited tendency of centrioles to form cilia, rather than structures necessarily performing highly specialized functions”. The widely held view that these cilia were of little consequence was largely based on the observations that they lacked the central microtubule pair that is a feature of motile cilia. The early 2000s saw renewed interest in mammalian primary cilia and the emergence of a new hypothesis that primary cilia are key signaling cellular organelles that function throughout the body (for review see: Singla and Reiter, 2006; Goetz and Anderson, 2010). With the discovery of new roles for primary cilia in regulating neural stem cell proliferation and migration in embryonic and adult brain regions (e.g., Breunig et al., 2008; Han et al., 2008; Spassky et al., 2008; Han and Alvarez-Buylla, 2010; Baudoin et al., 2012; Higginbotham et al., 2012, 2013; Guo et al., 2015), this hypothesis has triggered new studies designed to understand the function of primary cilia in brain tumors.

Primary cilia are now postulated to be involved in the pathogenesis of various cancers including brain tumors such as medulloblastoma (Han et al., 2009) and choroid plexus tumors (Li et al., 2016); however, the role(s) that these important organelles have in the pathogenesis of glioma, the most common form of brain cancer in adults, are just now beginning to be examined (also see Alvarez-Satta and Matheu, 2018). In this review we focus primarily on the most aggressive form of glioma, glioblastoma (GBM) and discuss the prevalence of ciliated cells in these tumors, the roles that these cilia may play in controlling glioma tumorigenesis, and findings that suggest the ciliated state of these cells may affect their susceptibility to standard of care GBM (glioblastoma) therapies.

## Prevalence of Primary Cilia in Glioma Biopsies and Cell Lines

A first step toward understanding the functions of primary cilia in tumors is to document the prevalence of these organelles in tumor tissues. For a summary of the status of cilia expression and function in non-glioma cancer subtypes, the reader is referred to a recent review (Eguether and Hahne, 2018). Our group has examined GBM biopsies collected from over 20 patients and have detected primary cilia in all biopsies (Sarkisian et al., 2014). The numbers of ciliated cells in these biopsies ranged from <1 to ∼25% of the population of tumor cells. Cilia were found on cells expressing Ki67, a cellular marker for proliferation, and on cells associated with the vasculature and pseudopalisading necroses, common pathological features of GBM. Closer examination of the cilia using EM analyses revealed the presence of normal appearing primary cilia and of cilia that appeared to have defects affecting the basal body or cilium. A mixture of normal and abnormal cilia in GBM biopsies was also reported by Moser et al. (2014) who examined 7 patient biopsies and reported finding normal cilia in one biopsy and cilia possessing various ultrastructural abnormalities that affected the basal body or axoneme in the remaining 6 biopsies. It is not clear whether these ultrastructural abnormalities affect cilia function. Currently, there are very few methods that allow assessment of cilia function. The best characterized is the sonic hedgehog (SHH) signaling pathway using SHH and subsequently monitor activation of downstream signaling genes (for review see: Goetz and Anderson, 2010). However, it should be noted that primary cilia also host many other signaling pathways including WNT, Notch, Hippo, platelet-derived growth factor (PDGF), insulin-like growth factor (IGF), mechanistic target of rapamycin (mTOR), and multiple G protein-coupled receptors (for review see: Liu et al., 2018; Wheway et al., 2018).

It is important to note that documentation of ciliated cells in human tumor biopsy samples could be significantly affected by the region of the tumor from which the biopsy is obtained, as well as the type of fixative used to preserve the sample and delays in fixation (e.g., Hua and Ferland, 2017). A typical research lab that receives a biopsy may know the general brain region from which the biopsy was obtained and details about the fixation of the tissue. In our experience, immunostaining of many cilia marker proteins in biopsy tissues is significantly improved if the tissues were immediately fixed in 4% paraformaldehyde. Clearly, additional analyses of GBM biopsies will be required to determine the relative frequency of ciliated cells within tumors and to improve the ultrastructural characterization of these cilia.

What cell types are ciliated in GBM tumors? The GBMmicroenvironment can contain multiple cell types that either resemble or derive from glia, microglia, oligodendrocytes, neurons, fibroblasts, and vascular cells (Charles et al., 2012; Rich, 2016). In our analyses of patient-derived xenograft (PDX) tumors, ciliated cells appear to be similarly, distributed in the large core of the tumor and in distal tumor satellite growths (Hoang-Minh L. B. et al., 2016). At this point, the distribution of primary cilia across the various cell types in human GBM *in situ* remains unknown. To determine which cells in GBM tumors are ciliated, it will be necessary to obtain and section larger regions of human brain that contain these tumors and then analyze the sections using combinations of immunohistochemistry, immuno-electron microscopy (EM), and reconstruction of serial sections.

*In vitro* analyses of tumor cell lines is a second approach used to study the role(s) that cilia might have in regulating tumor cell biology. Moser et al. (2009) performed the first immunocytochemical and quantitative EM analyses of various GBM cell lines (U-87 MG, T98G, U-251 MG, U-373 MG, and U-138 MG) and found that these cells rarely gave rise to cilia, or if the cells were ciliated, the cilia were often ultrastructurally abnormal. These particular GBM cell lines have fallen out of favor with many neurooncology researchers in part because the DNA profiles of the cell lines differ from those of the original tumor cells (Allen et al., 2016). It is unclear how these genetic changes might affect ciliogenesis. In view of this, we have studied ciliogenesis in five different recently derived human and mouse primary GBM cell lines and have found that approximately 5–30% of the cells across these cell lines were ciliated and that the cilia were ultrastructurally normal and stained positively for proteins known to localize to the ciliary axoneme and basal body (e.g., IFT88, ARL13B, SMO, GLI3, ADCY3, gamma and acetylated alpha tubulin, and PCM1; Sarkisian et al., 2014; Hoang-Minh L. et al., 2016; Hoang-Minh et al., 2018).

Can ciliogenesis be induced in GBM cells? Serum withdrawal is one way to induce differentiation and ciliogenesis (Santos and Reiter, 2008); however, we and others have been unable to stimulate ciliogenesis in cultured GBM cells using serum withdrawal (Moser et al., 2009; Sarkisian et al., 2014). These observations suggest that it may not be possible to induce ciliogenesis in glioma cells that if true may explain why many of the commonly used GBM cell lines studied *in vitro* typically lack cilia. Factors that may contribute to the low numbers of ciliated cells present in various cell lines, include structural cilia defects, the rapid turnover of the cultured cells, and heterogeneity of the cells with regard to their ability to generate or retain cilia. GBM growth is aggressive and so it is possible that the rapid turnover of cells within these tumors narrows the window of time during which cilia would be present. Alternatively, it may be that only a small fraction of cells in the tumor are capable of growing cilia. We examined this latter possibility by isolating cell clones from two PDX cell lines that normally display ∼10–25% ciliated cells at any given time and found that most of the clones that we isolated gave rise to ciliated progeny (Hoang-Minh L. B. et al., 2016). This finding indicates that even though ciliation was relatively low, most of the cells in these cell lines were capable of giving rise to ciliated daughter cells.

In summary, the consensus among GBM tumor biopsy and cell line studies indicates that anywhere from ∼<1 up to ∼30% of the cells in glioma biopsies and in these cell lines are ciliated at any given time. Future studies that characterize ciliated glioma lines should make reference, if possible, to the frequency of ciliated cells in the biopsy from which they were derived. If we are able to associate patient outcomes with the numbers and functions of ciliated cells within GBM tumor biopsies, then it may be possible use this information to better inform patient prognoses and treatments.

## Cilia and Gliomagenesis

Cilia are organelles typically associated with differentiated cells but are also assembled by dividing cells. In dividing cells, cilia are assembled by the mother centriole during G1 and can persist throughout the cell cycle but disappear during mitosis (Ford et al., 2018). Because cilia are intimately involved in cell division, it is possible that mutations that disrupt ciliogenesis could promote tumorigenesis as a result of a loss of cell cycle control (Plotnikova et al., 2008; Basten and Giles, 2013). In this section we will briefly review research data that support diametrically opposed roles for cilia in controlling tumor cell proliferation in glioma.

Recent studies of the lysophosphatidic acid receptor 1 (LPAR1) and cell cycle-related kinase (CCRK) and its substrate, intestinal cell kinase (ICK), suggest that proliferation of normal astrocytes and glioma cells is enhanced in cells that have either lost or have not synthesized primary cilia. The cilia of normal human astrocytes contain elevated levels of the LPAR1 (Loskutov et al., 2018), a receptor whose downstream signaling cascade activates the G-protein, Gα12/ Gαq (Goldsmith et al., 2011). Loskutov et al. (2018) found that proliferation can be induced in immortalized human astrocytes lacking primary cilia by activating LPAR1 signaling, signaling that is normally limited in ciliated cells because Gα12 and Gαq are excluded from the cilium. The increase in proliferation of the deciliated cells was found to be due to redistribution of LPAR1 to the plasma membrane of the cell where it was able to actively signal through association with Gα12/ Gαq. Furthermore, they found that treatments of deciliated astrocytes and of intracranial tumors in a mouse model of GBM with a small molecule inhibitor of LPA signaling significantly reduced proliferation of the astrocytes and growth of the intracranial GBM tumors, respectively. The results of this study support the idea that one function of primary cilia is to limit GBM proliferation, and that loss of tumor cell cilia may lead to the redistribution of LPAR1 to the plasma membrane and lead to cell proliferation as a result of increased LPAR1 signaling.

In addition to LPAR1 signaling, CCRK and its substrate, ICK, have been linked to the regulation of ciliogenesis and proliferation of tumor cells (Tian et al., 2012). Yang et al. (2013) found that knockdown of CCRK in cultured U251 GBM cells increased the numbers of ciliated cells in the cultures from ∼2 to 8% and slowed proliferation of the cells. They also found that depleting CCRK in NIH3T3 fibroblast cells increased levels of ICK at the tips of the cilia and prevented the cells from re-entering the cell cycle. Our group has also found that blocking ciliogenesis in one PDX tumor cell line by expressing a dominant negative form of Kif3a in the cells accelerated proliferation of these cells and their tumorigenic capacity in a PDX model (Hoang-Minh L. B. et al., 2016). Collectively, the results of these studies indicate that deciliation of transformed GBM cells may lead to loss of control of the cell cycle and increased tumor growth.

Interestingly, there is evidence that tumor cells may actively repress ciliogenesis thereby promoting tumor growth. For example, an increase in the levels of the transcription factor EZH2 in melanoma cells, which normally suppresses expression of genes regulating ciliogenesis, can lead to WNT/β-catenin-mediated tumorigenesis (Zingg et al., 2018). Notably, inhibition of EZH2 in GBM with elevated levels of EZH2 has been reported to suppress growth of these tumors (Jin et al., 2017). Thus, future studies should examine how EZH2 affects ciliogenesis in GBM.

There are also studies that suggest that cilia may promote tumor cell proliferation. Cells throughout the brain are ciliated (Fuchs and Schwark, 2004; Bishop et al., 2007; Guemez-Gamboa et al., 2014; Sarkisian and Guadiana, 2015) and it appears that cilia on progenitor cells may activate distinct signaling pathways that regulate cell proliferation. For example, in the hippocampal dentate granule zone of developing and adult brain, the cilia of neuronal progenitor cells mediate SHH signaling thereby promoting neurogenesis (Breunig et al., 2008; Han et al., 2008). Interestingly, a significant number of gliomas also respond to SHH (Dahmane et al., 2001; Bar et al., 2007; Clement et al., 2007; Gruber Filbin et al., 2013; Morgenroth et al., 2014). In a study of a PDX cell line reported to be responsive to SHH, we found that SHH only promoted proliferation of the tumor cells if they were ciliated or were capable of forming cilia, and that proliferation was blocked if ciliogenesis was inhibited using a dominant negative form of KIF3a or if SMO function in cilia was inhibited using cyclopamine (Hoang-Minh L. B. et al., 2016). We also found that CRISPR/Cas9-mediated suppression of PCM1, a protein that localizes to centriolar satellites and is required for ciliogenesis, inhibited the proliferation of two GBM cell lines apparently through enhancement of apoptotic cell death (Hoang-Minh L. et al., 2016).

Glioblastoma cells may also release factors from their cilia that could influence tumorigenesis. Recently, we used piggyBac transgenesis to label GBM cilia with Arl13b:GFP that allowed us to use live imaging to monitor the cilia (Hoang-Minh et al., 2018). We observed that a small fraction of GBM primary cilia released vesicles from their distal tips. We also found that culture media conditioned by ciliated GBM cells enhanced cell proliferation of GBM cells while media conditioned by cultures of GBM cells lacking cilia did not, an observation that suggests cilia themselves may release factors that enhance cell proliferation. The cells that appeared to release ciliary vesicles were Ki67^-^ suggesting that quiescent glioma cells may release factors into the microenvironment that could influence tumor growth. Our findings are noteworthy because they are consistent with recent reports that show primary cilia tip excisions occur in multiple non-cancer cell lines and that these excision events appear to be a mechanism that can drive quiescent cells back into the cell cycle (Nager et al., 2017; Phua et al., 2017; Ford et al., 2018). These observations indicate that examination of the content and the mitogenic activities of glioma-derived primary cilia vesicles may provide insight into mechanisms underlying tumor growth or recurrence.

## Cilia and Resistance of Tumors to Therapy

The standard of care therapies for GBM are surgery, temozolomide (TMZ) chemotherapy, and gamma-irradiation. Very little is known about how the presence or absence of cilia on glioma cells affects the efficacies of these treatments. We found that some ciliated cells express ZEB1, a transcription factor that promotes glioma initiation, invasiveness, and resistance to TMZ chemotherapy (Siebzehnrubl et al., 2013; Sarkisian et al., 2014). Using two PDX cell lines in which we inhibited cilia formation using either CRISPR/Cas9 ablation of either PCM1 or KIF3a, or of both, we found that the loss of either KIF3A or PCM1 in these cells was associated with an increase in the cell’s sensitivity to TMZ exposure (Hoang-Minh L. et al., 2016). Depletion of both KIF3A and PCM1 did not increase the sensitivity of the cells to TMZ above that observed in cells in which only one protein was ablated. These results suggest that cilia signaling may contribute to the survival of cells exposed to TMZ chemotherapy. Additional studies will be needed to determine whether this phenomenon is consistently observed in ciliated PDX cell lines and in intracranial models of GBM.

The results of a recent study of multiple types of drug-resistant cancer cell lines (e.g., rhabdoid tumor, non-small cell lung carcinoma, and lung adenocarcinoma) suggest that drug resistance in these cell lines is accompanied by an increase in ciliogenesis and cilia signaling as measured by SHH pathway activation (Jenks et al., 2018). These authors found that increased cilia length was sufficient to confer drug-resistance and that blocking either ciliogenesis or cilia signaling pathways was able to reverse drug-resistance. It would be worthwhile to determine whether these mechanisms are active in GBM cells. It is noteworthy that stimulation of ciliogenesis in cultured U251 GBM cells by knocking out CCRK also promoted the localization of members of the SHH pathway to cilia, an effect that could possibly enhance the signaling capacity of these structures (Yang et al., 2013). Similarly, we reported that Arl13b overexpression promotes SMO localization to GBM cilia in the absence of SHH, and that high levels of ARL13B and SMO expression in the tumors of glioma and gastric tumor patients was positively correlated with shortened post-diagnosis survival in these patients (Shao et al., 2017; Hoang-Minh et al., 2018). Thus, it is possible treatments or conditions that stimulate glioma cell ciliogenesis could induce drug resistance in these cells and lead to tumor recurrence.

## Summary and Future Directions

**FIGURE 1:**
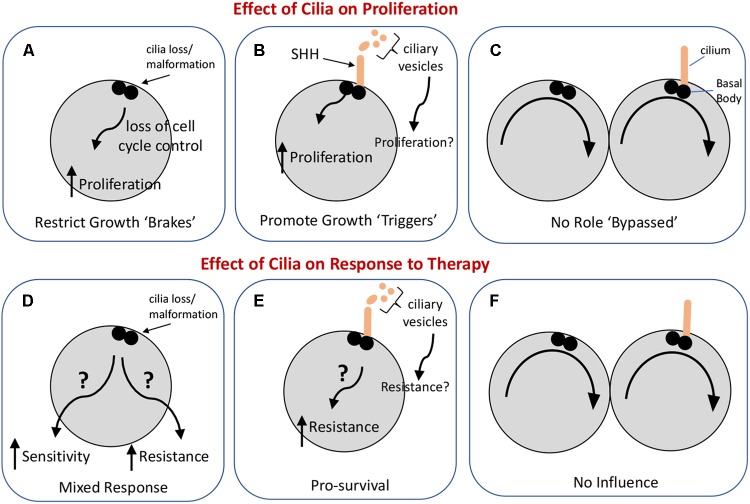
Possible influences of primary cilia on glioma cell proliferation and the response of these cells to standard-of-care therapies. The upper panels illustrate three ways in which cilia could impact glioma cell proliferation. **(A)** Loss or malformation of the cilium could alter signaling pathways in the cell leading to dysregulation of the cell cycle. **(B)** Cilia signaling may actively promote cell proliferation (e.g., in response to SHH) or may, through vesicle release, enhance tumor growth, and proliferation. **(C)** Cilia may not have an impact on cell proliferation. The lower panels illustrate three ways cilia could influence the response of glioma to therapy with the caveat that the type of therapy, e.g., chemotherapy versus irradiation, may shape the cell’s response differently. **(D)** Loss or malformation of the cilium could have one of two very different effects: it could increase the sensitivity of the cell to therapy under conditions in which cilia normally support cell survival, or it could increase the resistance of the cell to therapy if cilia-dependent signaling normally activates cell death pathways. **(E)** Cilia signaling could increase cell resistance to therapy by triggering cell survival pathways through either autocrine or paracrine signaling. **(F)** Glioma cells may have evolved so that their response to therapy occurs independently of cilia.

Taken together, the roles or primary cilia in GBM may depend on the relative abundance of ciliated cells in these tumors and the signaling capacities of the cilia. So far, data from existing experiments indicate that glioma cilia may have dual roles, either restricting or promoting gliomagenesis (Figure 1). If we can obtain high quality tumor biopsies, it may be possible to determine the numbers of ciliated cells in specific tumors and what signaling pathways may be active within these tumors. The question is whether this information could be useful in predicting how GBM tumors would respond to therapies employing SHH pathway inhibitors or chemotherapy/radiation. The hypotheses raised in this review require further testing in PDX models. Identification of signaling pathways activated by glioma cell cilia may point to new strategies to slow or inhibit the aggressive growth of these tumors.

## Author Contributions

MS and SS-R wrote the manuscript.

## Conflict of Interest Statement

The authors declare that the research was conducted in the absence of any commercial or financial relationships that could be construed as a potential conflict of interest.
